# Analysis on EZH2: mechanism identification of related CeRNA and its immunoassay in hepatocellular carcinoma

**DOI:** 10.1186/s12920-023-01594-9

**Published:** 2023-08-25

**Authors:** Haoran Zhao, Haishi Liu, Wenli Kang, Chao Zhan, Yingchun Man, Tong Qu

**Affiliations:** 1 Department of Hepatobiliary and Pancreatic Surgery, Affiliated Cancer Hospital of Harbin Medical University, Harbin, Heilongjiang Province 150040 China; 2 Department of Oncology, Beidahuang Industry Group General Hospital, No. 235 Hashuang Road, Harbin, Heilongjiang Province 150088 China

**Keywords:** ceRNA, EZH2, Bioinformatics, hepatocellular carcinoma, Immunoassay

## Abstract

**Objective:**

To screen the possible potential signaling pathways related to enhancer of zeste homolog 2 (EZH2) based on ceRNA mechanism, and to analyze the correlation between E2H2 and depths of various immune cell infiltration depths. The relationship between different immune checkpoints were also analyzed.

**Methods:**

First, the expression of EZH2 in pan-cancer (18 malignancies) was analyzed with the TCGA database. Hepatocellular carcinoma (HCC) tissues of 374 cases and normal tissues of 50 cases were analyzed in terms of the differential expression, overall survival (OS) and progression-free-survival (PFS). Then, we conducted GO and KEGG enrichment analysis on target gene. We also analyzed mRNA-miRNA and MicroRNA (miRNA)- long non-coding RNA (lncRNA) correlation with starbase databse, so as to determine the potential ceRNA mechanism associated with EZH2. Finally, immunoassay and drug-sensitivity analysis of EZH2 was performed.

**Results:**

Seven potential EZH2-related ceRNA pathways were screened out, namely lncRNA: Small Nucleolar RNA Host Gene 1 (SNHG1), SNHG 3, and SNHG 6-miR-101-3p-EZH2; and lncRNA: Long Intergenic Non-Protein Coding RNA 1978 (LINC01978), SNHG12, Ring Finger Protein 216 Pseudogene 1 (RNF216P1), and Coiled-coil Domain Containing 18 Antisense RNA 1 (CCDC18-AS1)-let-7c-5p—EZH2. Finally, 4 potential EZH2-related ceRNA pathways were identified through qPCR.According to immune correlation analysis, EZH2 may be positively correlated with T cells follicular helper, T cells Cluster of differentiation (CD)4 memory activated, Macrophages M0, and B cells memory (*P* < 0.05, cof > 0.2); while be negatively correlated with T cells CD4 + memory resting (*P* < 0.05, cof < -0.2). And EZH2 is positively correlated with Programmed Cell Death 1 (PDCD1) (*R* = 0.22), CD274 (*R* = 0.3) and Cytotoxic T-Lymphocyte Associated Protein 4 (CTLA4) (*R* = 0.23). According to drug sensitivity analysis, patients in the high expression group were more susceptible to the effects of various drugs including Sorafenib, 5-Fluorouracil, Doxorubicin, Etoposide, Paclitaxel, and Vinorelbine than those with low expression.

**Conclusion:**

This study revealed seven potential pathways of Enhancer of Zeste Homolog 2 (EZH2)-related ceRNA mechanisms: lncRNA (SNHG3, 6) -Mir-101-3P-ezh2; lncRNA (SNHG12, RNF216P1)-let-7c-5p—EZH2. We also analyzed the immunity and drug sensitivity of EZH2. Our study proves that EZH2 still has great research prospects in HCC.

**Supplementary Information:**

The online version contains supplementary material available at 10.1186/s12920-023-01594-9.

## Introduction

Primary liver cancer is the third leading cause of cancer death, and the incidence of HCC accounts for 80% of primary liver cancer. The major pathogenic causes of HCC are hepatitis (e.g., hepatitis B virus [HBV], hepatitis C virus [HCV], etc.), alcoholism, smoking, obesity, and congenital inheritance. There were approximately 906,000 new cases of liver cancer and 830,000 deaths worldwide in 2020 [1]. The advent of immunosuppressant has extended the window period for effective HCC treatment to a certain extent, but this does not mean the ultimate solution for HCC treatment. Perhaps only by further exploring effective biological therapeutic targets and on this basis combined with research in more tumor-related fields, including immunology, is the right way out for oncology research.

LncRNAs have been misunderstood over the past few decades. Since hardly no lncRNAs are expressed as proteins, they were once considered junk. It was not until this century that the role of lncRNA was gradually explored by researchers and became particularly popular in the recent decade. Even though lncRNAs do not express proteins at last, they still play an important role in many life activities such as Dosage compensation effect, epigenetic regulation, cell cycle regulation and cell differentiation regulation [2]. Abnormal regulation of the above processes will cause changes in proto-oncogenes and tumor suppressor genes, and eventually lead to the “alienation” of normal tissues.

**Fig. 1:**
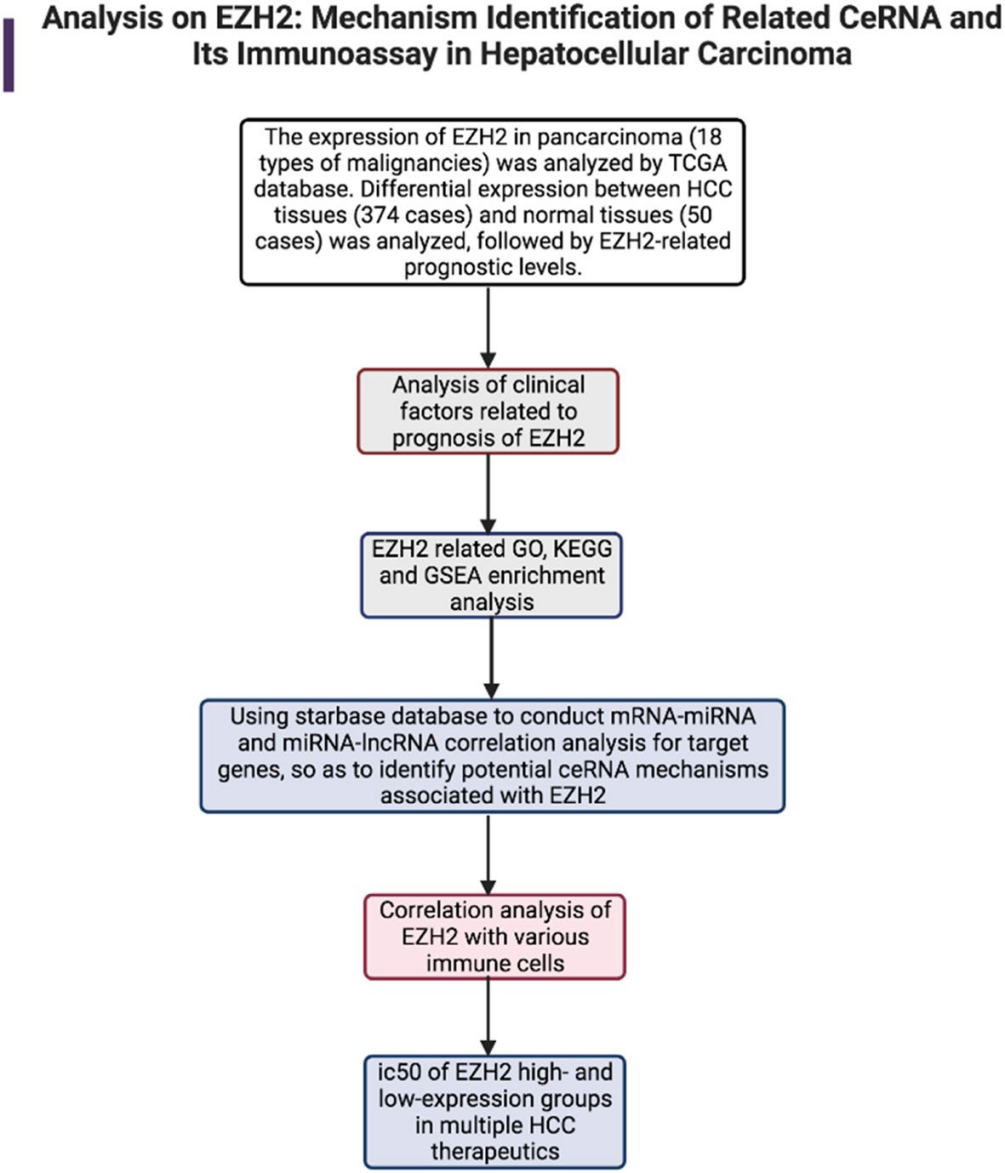
Research workflow

As the core component of the Polycomb repression complex 2 (PRC2), EZH2 conventionally catalyzes H3K27me3 as a histone methyltransferase, thereby repressing target gene transcription [3]. EZH2 is highly expressed in most solid tumors, including HCC, and plays a critical role in cell cycle regulation [4], PD-L1 expression [5], and chemo sensitivity [6]. This also stimulates our interest in further study of EZH2. Therefore, we took EZH2 as the research target, and analyzed miRNAs and lncRNAs that potentially correlated with ceRNA. The correlation between EZH2 and immune infiltration levels of related immune cells was also analyzed. We hope to find new therapeutic targets to provide ideas and references for future research. Our research workflow is shown below (Fig. 1).

## Methods

### EZH2-related differential analysis, prognostic analysis and protein expression

The R language package including the ggpubr was utilized to analyze the differential expression of E2F1 in 18 malignant tumors and plot the differential expression of EZH2 in pan-cancer. According to the magnitude of the *P* value of EZH2 in different cancer types, we divided samples into groups with *P* < 0.001, *P* < 0.01, and *P* < 0.05. The differential expression analysis of the target gene EZH2 was analyzed and visualized by the “limma”, “ggplot2” and “ggpubr” packages, and the prognosis-related survival curves were drawn by the “survival” and “survminer” packages. (*P* < 0.05 was defined as significant statistical difference.) Protein expression data were obtained through the HPA online database (https://www.proteinatlas.org/).

### Analysis of prognostic factors

The correlation between target gene expression and each prognostic factor was analyzed and visualized by “limma” and “ggpubr” packages. Heatmaps for each clinical prognostic factor were drawn with the “limma” and “ComplexHeatmap” packages. ROC curve, calibration curve and nomogram were obtained by “survival”, “survminer”, “timeROC”, “regplot”, and “rms” analysis. Univariate and multivariate COX analysis and forest plots were obtained by the “survival” package. *P* < 0.05 was defined as statistically significant difference.

### GO, KEGG and GSEA analysis

GO, KEGG and GSEA enrichment analysis were conducted and visualized by R language programs “clusterProfiler”, “org.Hs.eg.db”, “enrichplot”, “ggplot2”, “circlize”, “RColorBrewer”, “dplyr”, and “ComplexHeatmap” with 0.05 as *P* value filter. *P* < 0.05 was defined as statistically significant difference.

### Construction of potential signaling pathways based on ceRNA mechanism and analysis of miRNA and lncRNA survival

The mRNA-miRNA, miRNA-LncRNA interaction data were downloaded from the starbase database (https://rnasysu.com/encori/index.php). Among them, programNum  ≥ 2 was used as one of the mRNA-miRNA screening criteria. Correlation coefficient plot (*R* > 0.2 was defined as positive correlation, while *R* < -0.2 as negative correlation), differential expression (*P* < 0.01 was defined as statistical difference) and survival curve (*P* < 0.05 was defined as statistical difference) were screened and plotted by the R language package including “limma”, “reshape2”, “ggpubr”, “ggExtra”, “survival” and “survminer”. The diagram of potential EZH2-related ceRNA mechanism was drawned with BioRender software.

### Correlation analysis of EZH2 and immune cell infiltration

The correlation between EZH2 and various immune cells, immune checkpoints and immunotherapy analysis were visualized by R language packages including “limma”, “reshape2”, “ggplot2”, “ggpubr”, “vioplot”, “ggExtra” and “corrplot”. The data of immunotherapy analysis were obtained from the TCIA database (http://tcia.at/). The analysis of Programmed Cell Death Protein 1 (PDCD1), CD274 and CTLA4 that are related with EZH2 was conducted by the TIMER 2.0 GEPIA database (http://gepia.cancer-pku.cn), and the *P* value was calculated by Spearman statistical method. Among them, positive correlation was defined as *P* < 0.05 and Rp > 0.2; negative correlation was defined as *P* < 0.05 and *R* < -0.2; and *P* > 0.05 was defined as no significance.

### Drug sensitivity evaluation

We calculated the half maximal inhibitory concentration (IC50) of drugs by the R package with “pRRophetic” and its dependencies “car, ridge preprocessCore, genefilter, and sva”. The package included the information on the effects of 138 drugs. Boxplots were drawn using the R package “ggplot2”, and *P* < 0.05 was defined as statistically significant difference.

### RNA extraction, cDNA synthesis, and quantitative real-time PCR

RNA extraction and cDNA synthesis were performed using Trizol reagent (TaKaRa, Otsu, Japan) and the PrimeScript RT Reagent Kit with gDNA Eraser (Perfect Real Time) (TaKaRa, Otsu, Japan). Quantitative real-time PCR was performed using SYBR® Fast qPCR Mix (TAKARA), The relative expression of gene was normalized to GAPDH or U6 using 2^–ΔΔ*Ct*^ methods, respectively.

## Results

### Expression of EZH2 in pan-cancer and EZH2- related survival analysis

**Fig. 2:**
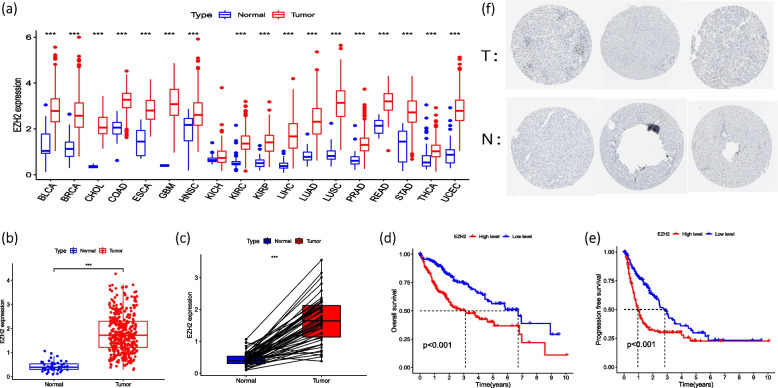
**a** Differential expression of EZH2 in pan-cancer between tumor groups and normal groups **b** Differential expression of EZH2 in HCC between tumor group and normal group **c** Pairwise differential analysis of EZH2 in HCC **d** Overall survival curve **e** Overall survival curve **f** Protein expression between tumor group and normal group in HPA database (T: tumor, N: normal) (* *p* < 0.05; ** *p* < 0.01; *** *p* < 0.001)

With the data of various cancer types downloaded from TCGA through UCSC Xena, which were analyzed by R language in terms of the expression of EZH2 in pan-cancer, it was found that EZH2 of 17 malignant tumor tissues (including HCC) was significantly differentially expressed compared with normal tissues (*P* < 0.001), as shown in Fig. 2a. Then, with the R package, we analyzed the differential expressions between HCC tumor group (374 cases) and normal group (50 cases), as shown in Fig. 2b and c. We also analyzed the disparity between EZH2 low-risk group and high-risk group in terms of disease free survival (DFS) and overall survival (OS). The results showed that EZH2 was differentially expressed between the HCC group and normal groups (TCGA normal and GTEx data) (*P* < 0.01), and the EZH2 low-risk group was significantly outperformed high-risk group in terms of both DFS (*P* < 0.001) and OS (*P* < 0.001), as shown in Fig. 2d and e. In addition, according to immunohistochemical staining test, EZH2 is significantly more stained in HCC as shown in Human Protein Atlas (HPA) (Fig. 2f).

### Analysis of prognostic factors of EZH2 in HCC

**Fig. 3:**
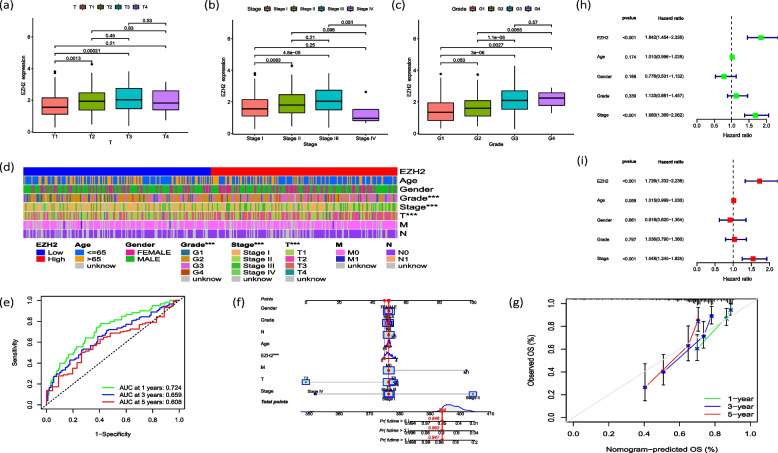
Prognostic factors: **a** T **b** Stage **c** Grade **d** Heat map **e** ROC curve **f** Nomogram **g** Calibration curve **h** Prognostic related risk factors identified by univariate COX regression analysis **i** Prognostic related risk factors identified by multivariate COX regression analysis (* *p* < 0.05; ** *p* < 0.01; *** *p* < 0.001)

Clinical correlation analysis showed that the expression of EZH2 was significantly different in T1 compared with in T2 and T3 (*P* < 0.01) (Fig. 3a); there was a significant difference between stageI, stageII, and stageIII (*P* < 0.01) (Fig. 3b); both grade1 and grade2 were significantly different from the other grades (Fig. 3c). And the classifications of T, stage and grade showed significant difference between the high and low expression groups (*P* < 0.001) (Fig. 3d). In addition, we drew the receiver operating characteristic (ROC) curve based on the target gene EZH2, which showed that the area under curve (AUC) at 1, 3, and 5 years were 0.724, 0.659, and 0.608 (Fig. 3e) respectively. We then built a gene correlation nomogram to evaluate the possibility that EZH2 can predict survival time in HCC (Fig. 3f), and validated the feasibility of the prediction method by a calibration curve (Fig. 3g). Finally, we concluded that EZH2 expression and HCC stage were independent risk factors for prognosis through univariate and multivariate COX regression analysis (Fig. 3h, i).

### EZH2-related Gene Set Enrichment Analysis (GSEA)

**Fig. 4:**
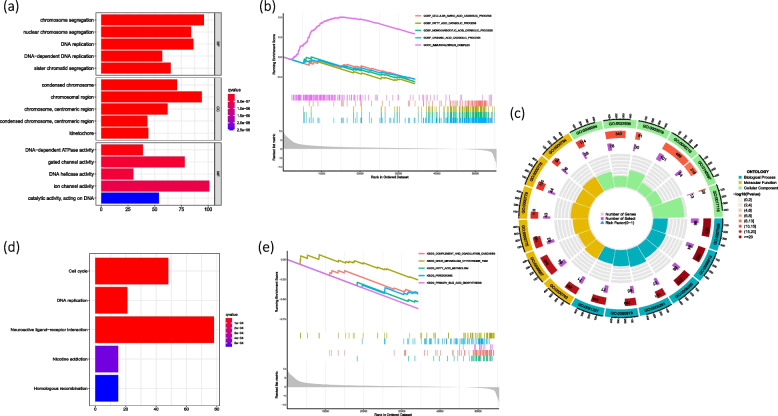
**a** GO enrichment analysis **b** GESA of GO **c** Circle diagram of GO enrichment analysis **d** GESA of KEGG **e** KEGG enrichment analysis

To elucidate the biological function of EZH2 in HCC, we analyzed the DEGs between the EZH2 low- and high-expressing groups according to the median EZH2 expression value (*P* < 0.001). GO analysis showed that: the top five enriched BPs were mainly related to biological processes such as chromosome segregation and DNA replication; the top five CCs mainly occurred in chromosomal, centromeric region and kinetochore; the top five MFs were mainly related to DNA-dependent ATPase activity, gated channel activity, DNA helicase activity, ion channel activity and catalytic activity, acting on DNA correlation (*P* < 0.001) (Fig. 4a, c). The GO-related GSEA pathway analysis showed that: the most enriched functions and pathways of patients with high EZH2 expression were immunoglobulin complex; while the low expression of EZH2 was associated with the catabolic process of cellular amino acid, fatty acid, monocarboxylic acid and organic acid (Fig. 3b). Meanwhile, in the KEGG enrichment analysis, the top 5 were mainly enriched in cell cycle, DNA replication, neuroactive ligand-receptor interaction, nicotine addiction and homotogous recombination (*P* < 0.001) (Fig. 4d). And low EZH2 expression was associated with complement and coagulation cascades, cytochrome P450 mediated drug metabolism, fatty acid metabolism, peroxisome and primary bile acid biosynthesis (Fig. 4e).

### Screening of signaling pathway of EZH2-related ceRNA mechanism

**Fig. 5:**
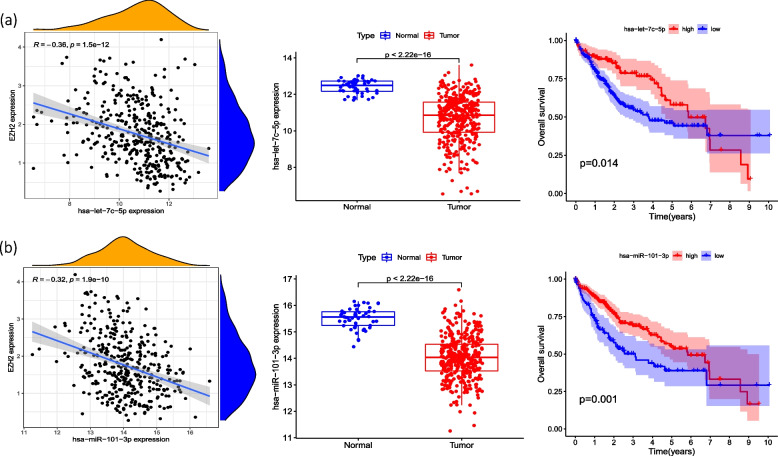
Correlation analysis, difference analysis and survival curve between two miRNAs and the target gene EZH2 **a** let-7c-5p **b** miR-101-3p

**Fig. 6:**
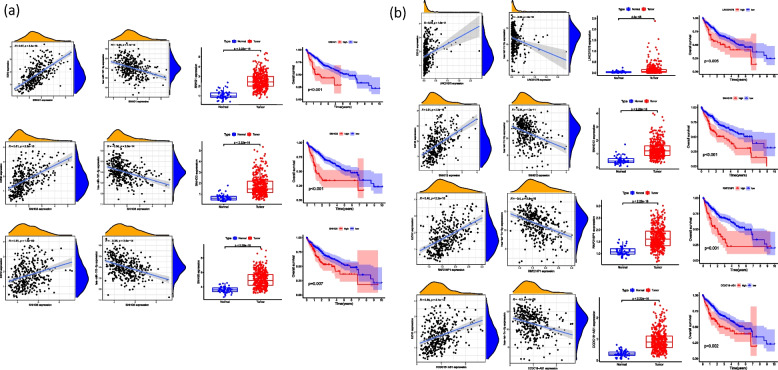
**a** Correlation, difference analysis and survival curve of miR-101-3p- EZH2-related lncRNAs with miRNA and EZH2, respectively; **b** Correlation, difference analysis and survival curves of let-7c-5p-ezh2-related lncRNAs with miRNA and EZH2, respectively

**Fig. 7:**
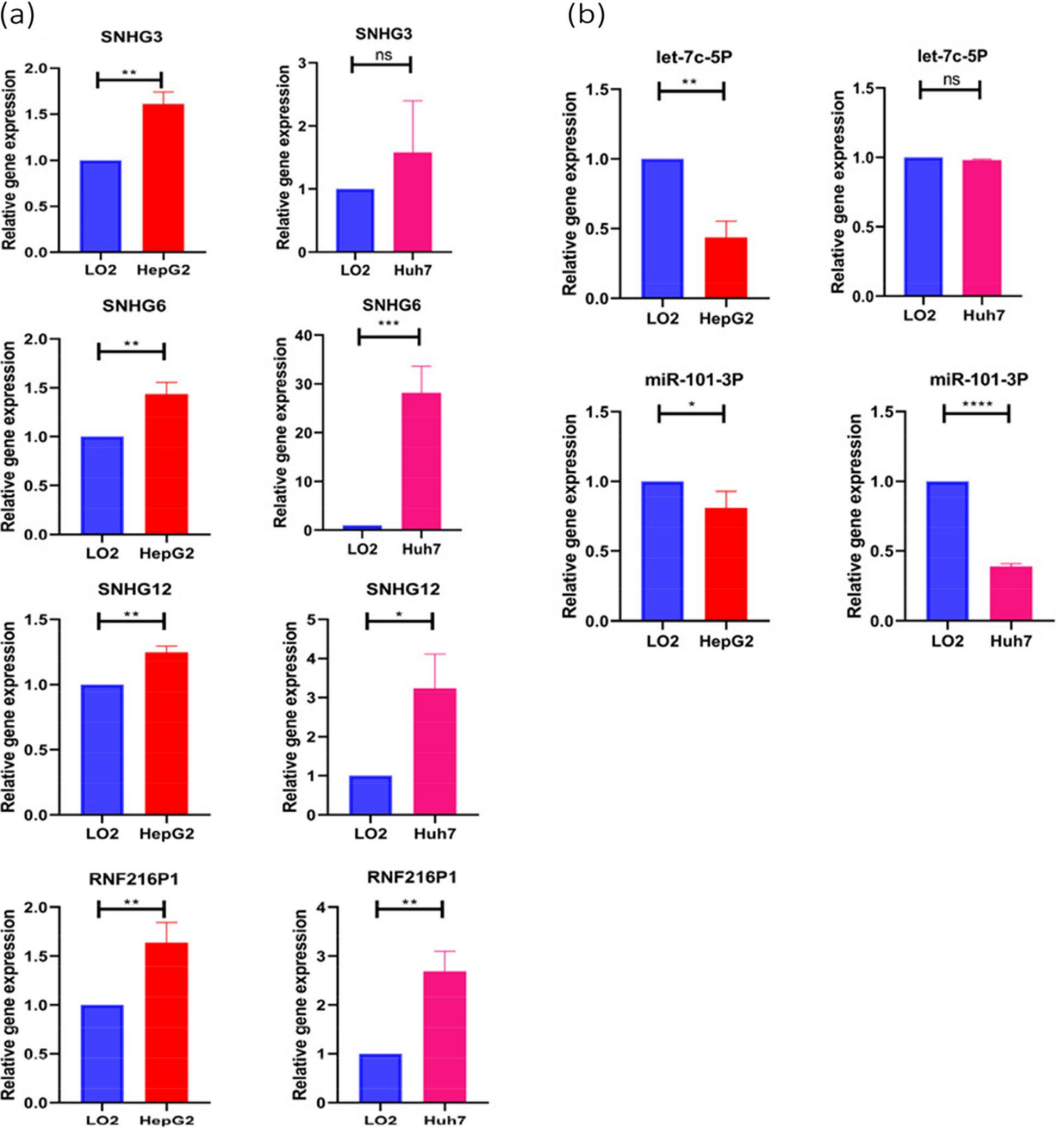
The verification of qPCR results of target gene

**Fig. 8:**
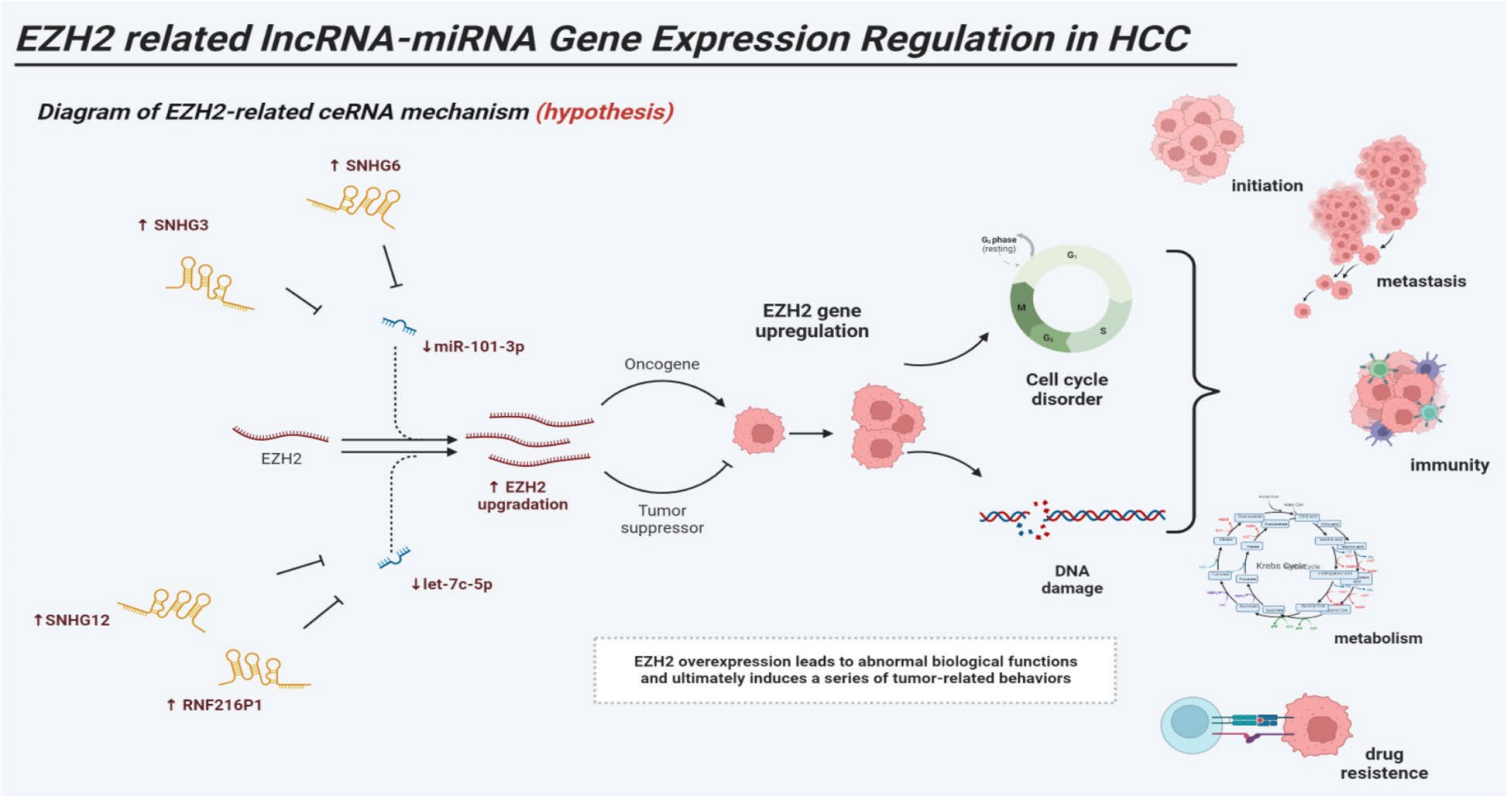
Diagram of EZH2-related ceRNA mechanism (hypothesis)

miRNAs that may have mRNA-miRNA interaction with EZH2 were searched out in the starbase database. And along with the search results with the original data of the TCGA database, we analyzed the correlation between these miRNAs and EZH2 (cor < -0.2, LogFC < 0, *P* < 0.05). Further screening was performed by examining the differential expression (*P* < 0.05) and survival curves (*P* < 0.05) of these miRNAs. Finally, two miRNAs were identified that may be involved in the ceRNA mechanism targeting EZH2, namely miR-101-3p and let-7c-5p (Fig. 4a, b). The lncRNAs that were related to the above two miRNAs, namely miR-101-3p and let-7c-5p, were screened out, especially ones that were positively correlated with EZH2 and meanwhile negatively correlated with the above two miRNAs. The screening was conducted with starbase database and by R language. Then the above lncRNAs were further analyzed and screened for their differential expression (*P* < 0.05) and survival curve (*P* < 0.05) (Fig. 5). Finally, a total of 7 signaling pathways that may have ceRNA mechanisms targeting EZH2 genes were found, namely lncRNA (SNHG1, SNHG3, SNHG6)-miR-101-3p-EZH2 (Fig. 6a), and lncRNA (LINC01978, SNHG12, RNF216P1, CCDC18-AS1)-let-7c-5p-EZH2. (Fig. 6b), We then verified the target gene, and the results showed that: The expression level of SNHG3 in hepatoma cell line HepG2 was significantly higher than that of normal liver cell line LO2 (*P* < 0.05), and the expression levels of SNHG6, SNHG12 and RNF216P1 in HepG2 and Huh7 hepatoma cell lines were significantly higher than that of LO2 (*P* < 0.05). The expression levels of miR-101-3p and let-7c-5p in hepatoma cell lines HepG2 and Huh7 were significantly lower than those of LO2 (*P* < 0.05). Among the screened pathways, lncRNA (SNHG3, SNHG6)-miR-101-3p-EZH2, and lncRNA (SNHG12, RNF216P1,)-let-7c-5p-EZH2 were preliminatively confirmed. It may be a potential EZH2-related signaling pathway with ceRNA mechanism (Fig. 7). To summarize these potential mechanisms, we drew a conceptual diagram of the EZH2-related ceRNA mechanism (Fig. 8).

### Analysis on EZH2 immune cells and checkpoint correlation

**Fig. 9:**
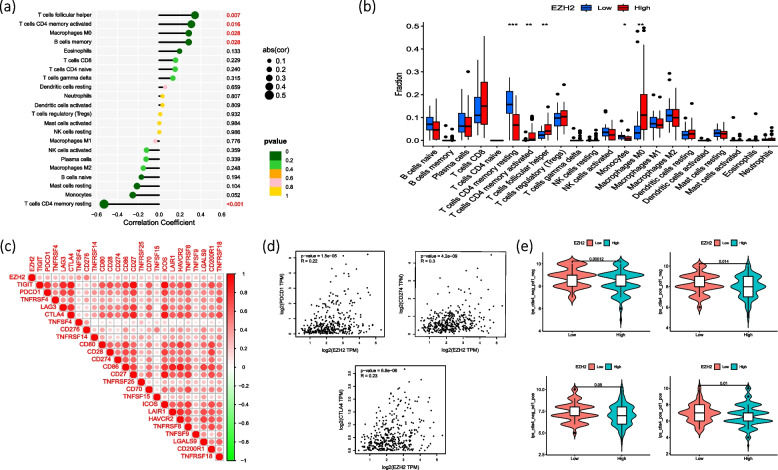
Analysis of the correlation between EZH2 and various immune cells. **a** lollipop chart **b** Boxplot **c** Correlation analysis between EZH2 and various immune checkpoints **d** Correlation of EZH2 with PDCD1, CD274 and CTLA4 in GEPIA database **e** The difference violin plot of PD-1, CTLA4 and the combined regimen in EZH2 high and low expression groups (* *p* < 0.05; ** *p* < 0.01; *** *p* < 0.001)

With the TIMER 2.0 database, we analyzed the correlation of infiltration among different types of immune cells, and the results showed that: EZH2 was probably positively correlated with T cells follicular helper, T cells CD4 memory activated, Macrophages M0, and B cells memory (*P* < 0.05, cof > 0.2); while it may be negatively correlated with T cells CD4 + memory resting (Fig. 9a, b). We also analyzed the correlation between EZH2 and various immune checkpoints (Fig. 9c). Then another analysis on the three immune checkpoints, namely PDCD1, CD274 and CTLA4, was performed with the GEPIA database. It showed that EZH2 was positively correlated with these three immune checkpoint (*R* > 0.2), as shown in Fig. 9d. In CTLA4, the results of PD-1 immunotherapy analysis showed that the EZH2 low expression group was significantly different from the high expression group when receiving anti-CTLA4 and anti-PD-1 combined with anti-CTLA4 treatment (*P* < 0.05) (Fig. 9e).

**Fig. 10:**
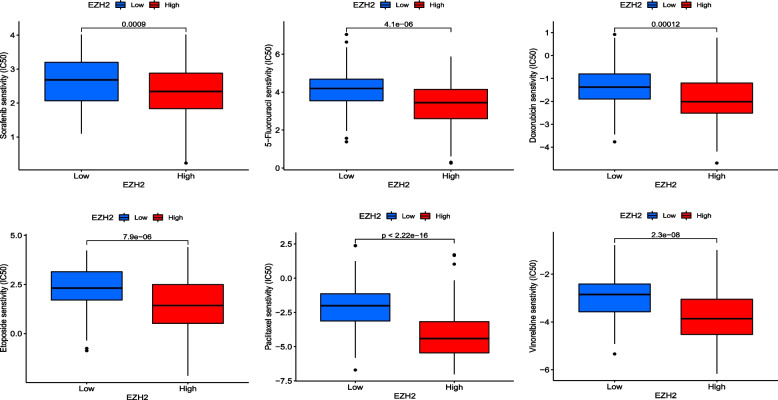
The ic50 of EZH2 high and low expression groups in various HCC treatment drugs (*P* < 0.001)

To further investigate the possibility of individualized treatment of EZH2 in HCC patients, we examined the relationship between risk score and IC50 of various drugs used in clinical treatment of HCC, including Sorafenib, 5-Fluorouracil, Doxorubicin, Etoposide, Paclitaxel, and Vinorelbine. In addition, we analyzed and listed the sensitivity of potential therapeutic agents. It showed that patients in the high-expression group appeared to be more susceptible to most drugs than those with low expression (Fig. 10).

## Discussion

As a member of the family of polycomb group genes (PcGs) [3], EZH2 is located on chromosome 7q35, covering a total of 20 exons and encoding 746 amino acid residues. It is composed of five domains, including 1) EED-interaction domain (EID); 2) Domain I and Domain II, 3) cysteine-rich domain (CXC domain); 4) C-terminal suppressor of variegation 39; and 5) enhancer of zeste and trithorax domain (SET domain). The histone methyltransferase activity of EZH2 is mainly maintained by the SET domain, which is also the central hub of the EZH2-related PRC2-dependent H3K27 methylation action mode. Gene silencing is mediated by PRC2, which is one of the PcG protein core complexes, mainly by regulating chromatin structure [7]. Under normal conditions, EZH2 mainly functions through three basic mechanisms: PRC2-dependent H3K27 methylation [3, 8], PRC2-dependent non-histone protein methylation [9] and PRC2-independent gene transactivation [10]. However, the abnormal expressed EZH2 will lead to the occurrence, development, invasion and metastasis of tumors. In recent years, a series of adverse chain reactions of malignant tumor progression due to EZH2 overexpression were reported more frequently, including malignant tumors like HCC [11], gastric cancer [12], breast cancer [13], esophageal cancer [14] and endometrial carcinoma [15]. Recent reports on HCC found a strong positive correlation between EZH2 and cycle-related genes such as CCND1, CDK2, CDK4, CCNB1, and CCNB2 [4]. And regulating the genes related to the above cells’ cycle results in HCC proliferation. Not only that, studies have shown that EZH2 can also inhibit the expression of the immune checkpoint inhibitor PD-L1 by directly upregulating the levels of CD274 and IFN regulatory factor 1 (IRF1) promoter H3K27me3 in liver cancer cells [5]. In addition, EZH2 also represses the tumor suppressor gene Atonal bHLH transcription factor 8 (ATOH8) by controlling DNA methylation and H3K27 methylation of its promoter, which in turn increases stem cell properties and reduces HCC chemo sensitivity [6]. The above findings all provide an important rationale for EZH2 as a therapeutic target for HCC, which motivates our in-depth research.

In terms of EZH2-related immune analysis, based on the TIMER database, different algorithms were chosen for the correlation of immune infiltration levels against different types of immune cells, so as to ensure the greatest accuracy and effectiveness. The results showed that: EZH2 may be positively correlated with T cells follicular helper, T cells CD4 memory activated, Macrophages M0, and B cells memory (*P* < 0.05, cof > 0.2); while EZH2 may be negatively correlated with T cells CD4 + memory resting (*P* < 0.05, cof < -0.2). Previous studies found that activated CD4 + T cells from HCCs stimulate CXCL10 production by macrophages, CXCL10 bind CXC chemokine receptor 3 on B cells and cause them to become IgG-producing plasma cells. IgG activated Fc receptor in macrophages to produce cytokines that reduce the anti-tumor immune response. Activation of CD4 + T cells in HCC stimulated macrophage production of CXCL10, and in turn, bind CXC chemokine receptor 3 on B cells and made them IgG-producing plasma cells. IgG activated Fc receptors in macrophages to produce cytokines that reduced antitumor immune responses [16]. This pathway witnessed the increased expression of DNA methyltransferase 1 and EZH2, which resulted from HCC and hepatoma cells. And reports about T cells follicular helper showed that EZH2 instructed the early commitment to TFH cell differentiation by stabilizing a cluster of TFH cell lineage-associated genes, including Bcl6 [17]. It was also confirmed the role of EZH2 in governing TFH cell differentiation by integrating phosphorylation-dependent Bcl6 activation and H3K27me3-dependent repression of p19Arf. Therefore, EZH2 regulates TH differentiation in a cell-type-specific manner [18]. In addition, a study of lymphoma pointed out that dysregulation of the GC, which result from constitutively active EZH2, activates lymphoma formation and identifies EZH2 as a possible therapeutic target for NHL and other GC-derived B-cell diseases. EZH2 maintains activation-induced cytidine deaminase function and prevents terminal differentiation of B cells in the germinal center, resulting in antibody diversification and affinity maturation. Dysregulation of the GC response by constitutively active EZH2 promotes lymphoma formation [19]. In addition to the above-mentioned reports on immune cells that were significantly different in our analysis, it was also found that phenotypic analysis of human CD8 + EZH2 + cells confirmed its greater effector capacity and the ability to reduce apoptosis sensitivity [20]. In the melanoma B16-tumor-bearing mice model of transferred Ezh2fl/flCd4Cre Pmel CD8 + T cells, the tumor suppressor effect of these cells was significantly reduced when EZH2 was insufficient [21]. EZH2 decreased CD8 + T cell suppression by inhibiting Tregs function. For instance, it promoted the frequency of CD8 + T cells producing ifn γ and reduced inhibitory marker expression on tumor-infiltrating T cells [22]. It has been reported that CAFs inhibited the expression of VASH1 by affecting the expression of VEGF and then upregulating the expression of EZH2 in HCC, thereby promoting the proliferation and angiogenesis of human umbilical vein endothelial cells (HUVECs). And this phenomenon has been further confirmed in vivo and in vitro experiments [23]. Furthermore, genetic deletion of EZH2 increased the expression of IL-15R, CD122 and NKG2D-activating receptors, resulting in enhanced NK cell production in hematopoietic stem and progenitor cells (HSPCs). And inhibition of EZH2 also improved mature NK cell function [24].

In this study, based on bioinformatics, we analyzed lncRNAs and miRNAs with potential ceRNA mechanisms targeting EZH2, namely miR-101-3p and three lncRNAs (SNHG1, 3, 6) that may interact with it, as well as hsa-let-7c-5p and 4 lncRNAs (LINC01978, SNHG12, RNF216P1, CCDC18-AS1) that may interact with it. Among the above ncRNAs, there are many miRNAs and lncRNAs that have been found closely related to the occurrence and development of tumors. In terms of miRNAs, a recent report showed that the expression of miR-101-3p in plasma exosomes from medulloblastoma (MB) patients was significantly higher than that of healthy controls, and these exosomal miRNAs could be delivered to tumor cells via exosomes. According to subsequent in vitro functional analysis, treatment of MB cells with the corresponding mimics of miR-101-3p significantly inhibited proliferation, colony formation, migration and invasion of tumor cells, and promoted apoptosis. Meanwhile, miR- 101-3p also targeted EZH2, thereby enhancing tumor suppression [25]. And it has been confirmed in several researches that SNHG6 inhibited apoptosis by regulating EZH2 expression via the sponging of MiR-101-3p in esophageal squamous-cell carcinoma [26, 27]. In addition, miR-101-3p also had coordinated or independent effects on the occurrence and development of malignant tumors such as breast cancer, [28] renal cell carcinoma [29] and oral squamous cell carcinoma [30]. Studies have shown that let-7c-5p enhanced 5-FU exposure (by inhibiting ABCC5/MRP5 expression) and co-targeted thymidylate synthase with 5-FU (let-7c reduced protein expression, 5-FU made the enzyme irreversibly inactivated). And this finally resulted in a strong synergistic effect in inhibiting the viability of hepatoma cells [31]. In terms of LncRNAs, SNHG1 repressed the transcription of Cyclin Dependent Kinase Inhibitor 1A (CDKN1A) and CDKN2B in the nucleus by enhancing EZH2-mediated H3K27me3 in the promoters of CDKN1A and CDKN2B, leading to de-repression of the cell cycle, thereby promoting HCC cell growth, cell cycle progression, metastasis and epithelial–mesenchymal transition (EMT) [32]. It has also been reported that SNHG3 promoted CD151 by inhibiting miR-128 signaling, resulting in HCC invasion, EMT, and sorafenib resistance [33]. SNHG12 regulated the SNHG12/miR-199a/b-5p pathway through a ceRNA mechanism, thereby reducing the inhibitory effect on Mixed Lineage Kinase 3 (MLK3, its expression was upregulated in tumor tissues and was associated with tumor progression and metastasis), thus enhanced expression of MLK3 and its downstream effectors in the NF-κB pathway, and finally promoted the occurrence and development of HCC [34]. Recent research reports suggest that CCDC18-AS1 was a lncRNA involved in pyroptosis which was related to the immune landscape of COAD patients [35].

In summary, 4 EZH2-related potential signaling pathways were screened out based on the ceRNA mechanism in multiple public databases and combined with R language analysis. In addition, we analyzed EZH2 drug sensitivity and the correlations between EZH2 and various immune cells as well as immune checkpoints were analyzed, which provided clear ideas and directions for future basic experimental research. However, it should be pointed out that although the above-mentioned signaling pathways and immune-related analysis have been verified in the previous literature, some of the results reached in this article were still in the stage of research and verification. We hope that this article can provide new research directions and biological targets for future HCC research.

## Conclusion

In conclusion, this study revealed four potential pathways of EZH2-related ceRNA mechanisms: lncRNA [SNHG3, SNHG6] -Mir-101-3P-ezh2; lncRNA (SNHG12, RNF216P1)-let-7c-5p—EZH2. We also analyzed the immunity and drug sensitivity of EZH2. At present, EZH2 still has great potential and prospect for the treatment of HCC. By further understanding its functional scope, we expect to find more potential therapeutic and diagnostic options for EZH2 in HCC.

### Supplementary Information

**Additional file 1: Supplement 1.** lncRNA-miRNA-mRNA pathway related parameters.

**Additional file 2: Supplement 2.** Other drug susceptibility evaluation.

## Data Availability

The raw data used in this experiment come from TCGA website (https://portal.gdc.cancer.gov/), starbase (https://rnasysu.com/encori/index.php), TIMER2.0 (http://timer.cistrome.org/), TCIA database (http://tcia.at/), KEGG pathway database (www.kegg.jp/kegg/kegg1.html), HPA online database (https://www.proteinatlas.org/) and GEPIA (http://gepia.cancer-pku.cn) (The relation of the three immune checkpoints associated with EZH2 by clicking on the link to the GEPIA home page, typing “EZH2” in the box, and then clicking “GoPIA”. Then click “correlation” and enter “EZH2” in “Gene A” and “CTLA4”, “PDCD1” and “CD274” respectively in “GENE B”. Then select “LIHC Tumor” in “TCGA Tumor” and finally click “Plot”). We would like to express our sincere thanks to the collectors and producers of the research data.
