# Supplementary material for: Analysis on EZH2: mechanism identification of related CeRNA and its immunoassay in hepatocellular carcinoma
Source: BMC Med Genomics. 2023 Aug 25;16:201. doi: 10.1186/s12920-023-01594-9 (PMC10463302; doi:10.1186/s12920-023-01594-9)
Supplement: Supplementary file 1 — Additional file 1: Supplement 1. lncRNA-miRNA-mRNA pathway related parameters. [file 12920_2023_1594_MOESM1_ESM.pdf]

.

Gene	miRNA	cor	pvalue	logFC	diffPval
EZH2	hsa-let-7c-5p	-0.358994232	1.48E-12	-1.731448191	2.08E-23
EZH2	hsa-miR-101-3p	-0.324907947	1.95E-10	-1.472341348	1.33E-24

lncRNA	Gene	cor	pvalue	logFC	diffPval
SNHG6	EZH2	0.309424841	1.46E-09	1.451472559	3.16E-21
LINC01748	EZH2	0.364661043	4.44E-13	0.214150227	5.86E-09
MKLN1-AS	EZH2	0.292765252	1.12E-08	0.321137059	7.04E-15
MIR3142HG	EZH2	0.332052101	5.66E-11	0.137124453	8.36E-05
THUMPD3-AS1	EZH2	0.46495335	0	0.698874601	6.85E-25
SNHG3	EZH2	0.508563563	0	1.07714845	3.52E-23
SNHG1	EZH2	0.668378444	0	1.457690034	4.47E-26
SNHG14	EZH2	0.282718232	3.61E-08	0.296678112	0.006128952
NUTM2B-AS1	EZH2	0.358641239	1.56E-12	0.171510007	1.07E-10
KCNMB2-AS1	EZH2	0.268647561	1.55E-07	0.552306808	1.26E-14

lncRNA	miRNA	cor	pvalue	logFC	diffPval
SNHG6	hsa-miR-101-3p	-0.381255267	3.63E-14	1.451472559	3.16E-21
LINC01748	hsa-miR-101-3p	0.212957203	3.63E-05	0.214150227	5.86E-09
MKLN1-AS	hsa-miR-101-3p	-0.249653019	1.25E-06	0.321137059	7.04E-15
MIR3142HG	hsa-miR-101-3p	-0.252726728	8.44E-07	0.137124453	8.36E-05
THUMPD3-AS1	hsa-miR-101-3p	0.268091785	1.84E-07	0.698874601	6.85E-25
SNHG3	hsa-miR-101-3p	-0.383187013	2.52E-14	1.07714845	3.52E-23
SNHG1	hsa-miR-101-3p	-0.352288558	4.09E-12	1.457690034	4.47E-26
SNHG14	hsa-miR-101-3p	-0.268967159	1.67E-07	0.296678112	0.006128952
NUTM2B-AS1	hsa-miR-101-3p	-0.239392071	3.43E-06	0.171510007	1.07E-10
KCNMB2-AS1	hsa-miR-101-3p	0.219008533	2.14E-05	0.552306808	1.26E-14

ncRNA	Gene	cor	pvalue	logFC	diffPval
KCNQ1OT1	EZH2	0.432840032	0	0.03030447	1.89E-13
LINC00641	EZH2	0.351035316	4.92E-12	0.228253498	1.05E-08
MIR4435-2HG	EZH2	0.19148672	0.00021667	0.994897371	9.95E-23
LINC01978	EZH2	0.34092798	1.60E-11	0.098140911	4.33E-05
NUTM2A-AS1	EZH2	0.300038794	4.67E-09	0.324726231	7.70E-14
LINC01234	EZH2	0.226732655	1.06E-05	0.315442936	2.96E-09
SNHG4	EZH2	0.432574798	2.65E-18	0.385498275	8.03E-19
SLC9A3-AS1	EZH2	0.222682731	1.62E-05	0.946115013	3.34E-13
TPT1-AS1	EZH2	0.16037268	0.001994026	0.146708089	8.15E-10
SNHG12	EZH2	0.512439848	0	0.807166246	7.10E-22
LMCD1-AS1	EZH2	0.289872188	1.35E-08	0.03362808	2.45E-08
MIR137HG	EZH2	0.380830905	3.24E-14	0.01302698	0.002784826
CYTOR	EZH2	0.254436423	7.72E-07	1.341885312	8.43E-21
RNF216P1	EZH2	0.483249971	0	0.559672548	4.16E-19
SNHG14	EZH2	0.282718232	3.61E-08	0.296678112	0.006128952
LINC00665	EZH2	0.351756464	4.42E-12	0.776322126	1.62E-10
LINC00632	EZH2	0.297139539	5.60E-09	0.062666304	0.015230628
VASH1-AS1	EZH2	0.281678681	4.07E-08	0.192794036	1.29E-05
ASH1L-AS1	EZH2	0.454066966	0	0.405162476	5.26E-18
CCDC18-AS1	EZH2	0.352904045	3.73E-12	0.616415635	6.26E-23

lncRNA	miRNA	cor	pvalue	logFC	diffPval
KCNQ1OT1	hsa-let-7c-5p	-0.221292557	1.83E-05	0.03030447	1.89E-13
LINC00641	hsa-let-7c-5p	-0.227389851	1.06E-05	0.228253498	1.05E-08
MIR4435-2HG	hsa-let-7c-5p	-0.361779082	9.59E-13	0.994897371	9.95E-23
LINC01978	hsa-let-7c-5p	-0.322776366	2.03E-10	0.098140911	4.33E-05
NUTM2A-AS1	hsa-let-7c-5p	-0.230950101	7.61E-06	0.324726231	7.70E-14
LINC01234	hsa-let-7c-5p	0.216537502	2.66E-05	0.315442936	2.96E-09
SNHG4	hsa-let-7c-5p	-0.290093476	1.32E-08	0.385498275	8.03E-19
SLC9A3-AS1	hsa-let-7c-5p	-0.226408579	1.15E-05	0.946115013	3.34E-13
TPT1-AS1	hsa-let-7c-5p	-0.305128046	2.49E-09	0.146708089	8.15E-10
SNHG12	hsa-let-7c-5p	-0.344360441	1.31E-11	0.807166246	7.10E-22
LMCD1-AS1	hsa-let-7c-5p	-0.214174672	3.27E-05	0.03362808	2.45E-08
MIR137HG	hsa-let-7c-5p	-0.211189659	4.22E-05	0.01302698	0.002784826
CYTOR	hsa-let-7c-5p	-0.38124437	3.64E-14	1.341885312	8.43E-21
RNF216P1	hsa-let-7c-5p	0.398209333	5.78E-16	0.559672548	4.16E-19
SNHG14	hsa-let-7c-5p	-0.246933081	1.64E-06	0.296678112	0.006128952
LINC00665	hsa-let-7c-5p	-0.25331277	8.66E-07	0.776322126	1.62E-10
LINC00632	hsa-let-7c-5p	-0.214003504	3.31E-05	0.062666304	0.015230628
VASH1-AS1	hsa-let-7c-5p	-0.256148792	6.48E-07	0.192794036	1.29E-05
ASH1L-AS1	hsa-let-7c-5p	-0.294032472	9.64E-09	0.405162476	5.26E-18
CCDC18-AS1	hsa-let-7c-5p	-0.301203906	4.05E-09	0.616415635	6.26E-23

Supplement 1：lncRNA-miRNA-mRNA pathway related parameters
