# Supplementary material for: Analysis on EZH2: mechanism identification of related CeRNA and its immunoassay in hepatocellular carcinoma
Source: BMC Med Genomics. 2023 Aug 25;16:201. doi: 10.1186/s12920-023-01594-9 (PMC10463302; doi:10.1186/s12920-023-01594-9)
Supplement: Supplementary file 2 — Additional file 2: Supplement 2. Other drug susceptibility evaluation. [file 12920_2023_1594_MOESM2_ESM.pdf]

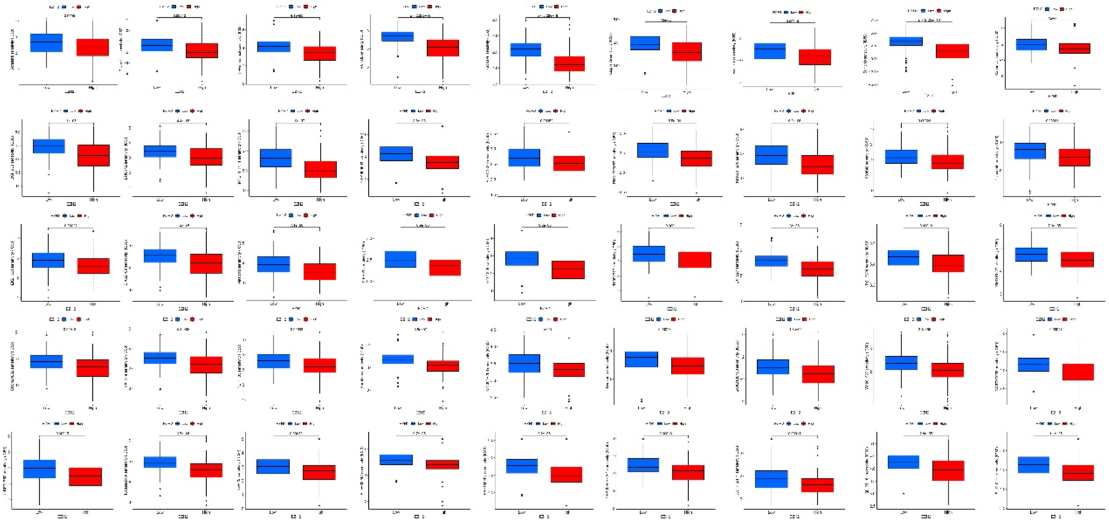

Supplement 2：other drug susceptibility evaluation
